# Healthcare use and costs in the last six months of life by level of care and cause of death

**DOI:** 10.1186/s12913-024-10877-5

**Published:** 2024-05-30

**Authors:** Yvonne Anne Michel, Eline Aas, Liv Ariane Augestad, Emily Burger, Lisbeth Thoresen, Gudrun Maria Waaler Bjørnelv

**Affiliations:** 1 Department of Health Management and Health Economics, Institute of Health and Society, University of Oslo, Oslo, Norway; 2 Faculty of Social Sciences, University of Applied Sciences Zittau/ Görlitz, Görlitz, Germany; 3 Division for Health Services, Norwegian Institute of Public Health, Oslo, Norway; 4 Center for Health Decision Science, Harvard T.H. Chan School of Public Health, Boston, MA USA; 5 Department for Interdisciplinary Health Sciences, Institute of Health and Society, University of Oslo, Oslo, Norway; 6 Department of Public Health and Nursing, Norwegian University of Science and Technology, Trondheim, Norway

**Keywords:** End-of-life, Healthcare use, Healthcare costs, Cause of death, Home- and community-based care, Modelling, Dementia

## Abstract

**Background:**

Existing knowledge on healthcare use and costs in the last months of life is often limited to one patient group (i.e., cancer patients) and one level of healthcare (i.e., secondary care). Consequently, decision-makers lack knowledge in order to make informed decisions about the allocation of healthcare resources for all patients. Our aim is to elaborate the understanding of resource use and costs in the last six months of life by describing healthcare use and costs for all causes of death and by all levels of formal care.

**Method:**

Using five national registers, we gained access to patient-level data for all individuals who died in Norway between 2009 and 2013. We described healthcare use and costs for all levels of formal care—namely primary, secondary, and home- and community-based care —in the last six months of life, both in total and differentiated across three time periods (6-4 months, 3-2 months, and 1-month before death). Our analysis covers all causes of death categorized in ten ICD-10 categories.

**Results:**

During their last six months of life, individuals used an average of healthcare resources equivalent to €46,000, ranging from €32,000 (Injuries) to €64,000 (Diseases of the nervous system and sense organs). In terms of care level, 63% of healthcare resources were used in home- and community-based care (i.e., in-home nursing, practical assistance, or nursing home care), 35% in secondary care (mostly hospital care), and 2% in primary care (i.e., general practitioners). The amount and level of care varied by cause of death and by time to death. The proportion of home- and community-based care which individuals received during their last six months of life varied from 38% for cancer patients to 92% for individuals dying with mental diseases. The shorter the time to death, the more resources were needed: nearly 40% of all end-of-life healthcare costs were expended in the last month of life across all causes of death. The composition of care also differed depending on age. Individuals aged 80 years and older used more home- and community-based care (77%) than individuals dying at younger ages (40%) and less secondary care (old: 21% versus young: 57%).

**Conclusions:**

Our analysis provides valuable evidence on how much healthcare individuals receive in their last six months of life and the associated costs, broken down by level of care and cause of death. Healthcare use and costs varied considerably by cause of death, but were generally higher the closer a person was to death. Our findings enable decision-makers to make more informed resource-allocation decisions and healthcare planners to better anticipate future healthcare needs.

**Supplementary Information:**

The online version contains supplementary material available at 10.1186/s12913-024-10877-5.

## Background

Healthcare resources—such as trained staff, equipment, and beds in hospitals and nursing homes—are limited; therefore, decisions about how to use available healthcare resources are inevitable in publicly funded healthcare systems. Ideally, decision-makers base their resource-allocation decisions on valid, comprehensive evidence and societal preferences which indicate what is most important to the recipients of healthcare services. In reality, decision-makers have to make high-impact decisions under conditions of great uncertainty. As a result, scarce healthcare resources may be used inefficiently, due to significant knowledge gaps about which patient group needs which healthcare resources at which level of care.

The last months of life are known to be ‘resource intensive’ [1–3]. Existing knowledge on resource use during the last months of life is fragmented and incomprehensive, with studies focusing on single parameters of care and patient groups most commonly diagnosed with a specific type of cancer [4–9]. We identified two major knowledge gaps in the existing literature on resource use and costs in the last months of life.

For the first knowledge gap, extant research on resource use in the last months of life has focused predominantly on secondary healthcare services provided at hospitals; data on the use of primary healthcare (i.e., general practitioners (GPs), emergency primary healthcare) and home- and community-based care (i.e., care institutions, home nursing) is harder to find. Only if healthcare planners are provided with knowledge about healthcare use and costs at all levels of care can they fully optimise priorities when planning for future care needs.

We are aware of a limited number of studies which report on resource use and costs beyond secondary care. A systematic review summarised healthcare use in the last months of life in 3.7 million adult cancer patients [10]. Langton and colleagues found that secondary care received in hospitals was reported in most of the studies, while components of community care, was mentioned in 41% of the studies and physician visits as an indicator of primary care was mentioned in only 30% of the studies [10]. Nevertheless, none of the included studies provided data for all levels of formal care simultaneously. Tanuseputro’s population-based study looked into healthcare costs in the last 12 months of life in Ontario in 2010–2013 [11]. This study provided evidence on costs in the last year of life broken down by healthcare sector: total costs in the last year of life consisted of an average of 43% spent on inpatient care, while physician services, medications/devices, laboratories, and emergency rooms contributing to less than 20% of total costs; almost 16% was spent on long-term-care in institutions, and approximately 8% was spent on home care [11]. However, the study did not report resource use by cause of death. Finally, a recent registry-based study from 2022 investigated care pathways for patients with different cancer diagnoses in the last six months of life for all levels of formal care [12]. The authors found that, depending on their type of cancer, patients utilised 44–66% of resources in secondary care and 31–52% in home- and community-based care during their last six months of life [12]. To our knowledge, comparable estimates for all levels of formal care are not available for causes of death other than cancer.

For the second knowledge gap, knowledge on resource use and costs in the last months of life is only available for a limited number of causes of death, such as circulatory diseases [13], stroke [14], and respiratory diseases [15]. Still, most of the available evidence is on cancer patients’ use of secondary care in the last months of life [5–10, 16, 17]. Far less is known about resource use for individuals dying with mental diseases like dementia and Alzheimer’s disease, with existing studies focusing solely on costs [18]. Healthcare planners in publicly funded healthcare systems cannot afford inefficient allocation of scarce resources for a large and fast-growing patient group like dementia: the WHO expects that 75 million individuals will suffer from dementia in 2030, with the number rising to 132 million in 2050 [19]. Thus, ageing societies worldwide have an urgent need for evidence on resource use and costs for progressive mental diseases like dementia.

We aim to address these knowledge gaps by estimating healthcare use and costs in the six last months of life for all levels of formal care—primary, secondary, and home- and community-based care—for all causes of death, for two age groups, and for three time periods before death. In doing so, we aim to provide a more complete understanding of resource use and costs in the last six months of life. Our findings will support decision-makers in making more informed decisions regarding resource allocation and healthcare planners in better anticipating future healthcare needs.

## Methods

In this study, we describe healthcare use at all levels of formal care (primary, secondary, and home- and community-based care) during the last six months of life of all individuals who died in Norway between 2009 and 2013. Using a healthcare perspective, we estimated the cost of healthcare during individuals’ last six months of life. To gather this information, we drew from five patient-level national registries.

### Healthcare in Norway

Norway’s healthcare system is built on the principles of universal coverage and egalitarianism: healthcare is provided based on need for treatment, regardless of a person’s socioeconomic status, ethnicity, or area of residence. Healthcare is publicly funded, primarily through taxes, and membership in the public health insurance is mandatory [20]. Norwegian municipalities organise primary and home- and community-based care. In primary care, GPs play an important role and function as gatekeepers, referring patients to specialised healthcare when necessary. GPs provide primary care during office hours and emergency primary healthcare outside office hours [20]. The guiding principle for home- and community-based care is enabling patients to stay at home for as long as possible but to move to care facilities (i.e., nursing homes) when needed. Four state-owned Regional Health Authorities are responsible for organising specialised secondary care; inpatient care is provided at hospitals, while outpatient treatments are provided both at hospitals and by self-employed specialists in private practice [20].

### Data

#### National registries

We retrieved data from The Norwegian Causes of Death Register (CDR) [21], The Norwegian Patient Register (NPR) [22], Norwegian Control and Payment of Health Reimbursements Database (KUHR) [23], The Individual-based Statistics for Nursing and Care Services Register (IPLOS) [24], and Statistics Norway (SSB) [25].

#### Causes of death

Our study population contained all decedents in Norway in between 2009 and 2013, drawn from CDR. From this registry, we retrieved information on cause of death, coded as an individual’s underlying cause of death using ICD-10 codes [21]. Data on underlying cause of death was based on an individual’s death certificate, which was completed by a physician. For example, if a cancer patient died from pneumonia, the physician reported pneumonia as the immediate cause of death and cancer as the underlying cause of death. Only one underlying cause of death per person is recorded, identifying the diagnosis that most contributed to the individual’s death. In dialogue with the registries, we agreed on the following categories of underlying cause of death: Communicable diseases (ICD-10 codes A00–B99), Cancer (C00–C97), Endocrine, nutritional, and metabolic diseases (E00–E99), Mental and behavioural diseases (F00–99), Diseases of the nervous system and sense organs (G00–H95), Diseases of the circulatory system (I00–99), Diseases of the respiratory system (J00–99), Diseases of the digestive system (K00–93), Injuries (V01–Y89), and Other diseases (L, M, N, O, P, Q, R, S, T and U). In Table 1, we list the five most common ICD-10 codes within each of the categories described above, providing the reader with an overview of which causes of death are represented in each category.

**Table 1:** Causes of death

Causes of death ICD-10	N % of entire population	Most common subgroups	ICD-10	N	%
Communicable	4845	Other sepsis	A41	1864	39%
A00-B99	2%	Other and unspecified infectious diseases	B99	1110	23%
		Other gastroenteritis and colitis of infectious and unspecified origin	A09	679	14%
		Other bacterial intestinal infections	A04	232	5%
		Erysipelas	A46	143	3%
Cancer	53,915	Malignant neoplasm of bronchus and lung	C34	10,766	20%
C00-C97	26%	Malignant neoplasm of colon	C18	5906	11%
		Malignant neoplasm of prostate	C61	5154	10%
		Malignant neoplasm of pancreas	C25	3375	6%
		Malignant neoplasm of breast	C50	3261	6%
EMD^1^	5147	Unspecified diabetes mellitus	E14	2208	43%
E00-E99	2%	Type 2 diabetes mellitus	E11	1006	20%
		Volume depletion	E86	502	10%
		Type 1 diabetes mellitus	E10	316	6%
		Unspecified protein-energy malnutrition	E46	251	5%
Mental	10,419	Unspecified dementia	F03	8074	77%
F00-99	5%	Mental and behavioral disorders due to use of alcohol	F10	878	8%
		Vascular dementia	F01	808	8%
		Depressive episode	F32	159	2%
		Mental and behavioral disorders due to multiple drug use and use of other psychoactive substances	F19	106	1%
Nervous	8394	Alzheimer disease	G30	3779	45%
G00-H95	4%	Parkinson disease	G20	1491	18%
		Spinal muscular atrophy and related syndromes	G12	616	7%
		Multiple sclerosis	G35	498	6%
		Other degenerative diseases of nervous system, not elsewhere classified	G31	407	5%
Circulatory	65,027	Acute myocardial infarction	I21	15,845	24%
I00-99	31%	Chronic ischaemic heart disease	I25	8375	13%
		Stroke, not specified as hemorrhage or infarction	I64	7296	11%
		Congestive heart failure	I50	7231	11%
		Atrial fibrillation and flutter	I48	3726	6%
Respiratory	20,395	Other chronic obstructive pulmonary disease	J44	9388	46%
J00-99	10%	Bronchopneumonia, unspecified	J18	7737	38%
		Other interstitial pulmonary diseases	J84	726	4%
		Emphysema	J43	469	2%
		Asthma	J45	453	2%
Digestive	6379	Alcoholic liver disease	K70	716	11%
K00-93	3%	Other diseases of digestive system	K92	660	10%
		Paralytic ileus and intestinal obstruction without hernia	K56	645	10%
		Vascular disorders of intestine	K55	462	7%
		Diverticular disease of intestine	K57	451	7%
Injury	12,729	Exposure to unspecified factor	X59	3286	18%
V01-Y89	6%	Intentional self-harm by hanging, strangulation and suffocation	X70	1170	17%
		Unspecified fall	W19	1113	13%
		Accidental poisoning by and exposure to narcotics and psychodysleptics [hallucinogens], not elsewhere classified	X42	821	9%
		Other fall on same level	W18	549	5%
Others	20,049	Other sudden death, cause unknown	R96	3068	25%
L, M, N, O, P, Q, R, S, T and U	10%	Senility	R54	2041	24%
		Other ill-defined and unspecified causes of mortality	R99	1606	23%
		Unspecified kidney failure	N19	1504	9%
		Other disorders of urinary system	N39	1453	5%

^*1*^ *EMD = Endocrine, nutritional and metabolic diseases*

### Healthcare use and costs

#### Primary care

When a patient receives primary healthcare in Norway, the provider sends a claim to The Norwegian Health Economics Administration (HELFO) [26]. These claims, their associated costs, and information on patient co-payments are entered into KUHR. We used information on treatments provided by GPs, either at the GP’s office or as emergency primary healthcare outside normal office hours. We present primary healthcare use as number of visits. Costs of primary care were also retrieved from KUHR.

#### Secondary care

For each secondary care treatment provided at a hospital in Norway the patient’s diagnosis and the treatment provided are registered in NPR, including information on whether inpatient or outpatient treatment was provided. All patient-related activity in hospitals is grouped into approximately 900 diagnosis-related groups (DRGs), which reflect the treatment provided and its associated mean cost across several hospitals which provide the treatment [27]. DRG costs include direct costs associated with the treatment of the disease, cost of complications during the hospital stays, and overhead costs. Additionally, we retrieved laboratory and radiology costs and patients’ co-payments from KUHR. We used information on all hospital inpatient (including day and overnight treatments) and outpatient treatments, number of days in the hospital, and total costs during the last six months of life as estimated by DRGs.

#### Home- and community-based care

All Norwegian municipalities must provide information to IPLOS [24]. We retrieved information on the number of days individuals spent in care institutions during their last six months of life. Additionally, we obtained information regarding whether individuals received home-based care in the form of practical or nursing assistance, which was measured in hours.

#### Healthcare costs

We have used a healthcare perspective and show the estimated costs in 2013 euros (€) using the 2013 annual exchange rate. All costs were estimated at patient level.

To estimate the costs of primary care services, we used information on reimbursement claims and patient co-payments which are recorded in KUHR for each GP consultation and emergency primary care visit. Costs were estimated by dividing the sum of claims and patient co-payments by 0.3. This is in line with recommendations from the Norwegian Directorate of Health, who estimated that all claims and co-payments recorded in KUHR reflect approximately 30% of the total cost of primary care [28]. Other guidelines suggest using 0.5 [29], but a recent study found that this resulted in an underestimation of actual costs [30].

Secondary care costs were estimated by multiplying DRG weights by the yearly unit price of a DRG weight. The costs of radiology and laboratory services are recorded in KUHR. Similarly to other KUHR estimates, we summed costs of radiology and laboratory services as well as patient co-payments and dividing the total cost estimate by 0.3 [28, 31]. We added these costs to the patient-level hospital costs.

To calculate costs of home- and community-based care, we multiplied days in care institutions by SSB’s official corrected gross operating expenses, published in KOSTRA (The Municipality- State- Reporting) [25]. To estimate the costs of practical and nursing assistance, we multiplied the number of hours of each type of care service that individuals received by the corresponding cost per hour, as estimated by Langeland and colleagues [31].

We estimated total healthcare costs by adding the costs in primary, secondary, and home- and community-based care. Variables of healthcare use and costs are detailed in Table 2. To estimate country-specific costs, the readers can multiply their country-specific unit costs by the healthcare use estimates for all decedents as presented in Table 2 and decomposed for all causes of death and by age (younger and older than 80 years) in the detailed Supplementary Material 1-3.

**Table 2:** Total and monthly average healthcare use and costs in € for all decedents, presented in three time periods before death

	6 to 4 months before death		3 to 2 months before death		1 month before death		Total six months before death	
	Monthly		Monthly				Total	
	Mean	s.e.	Mean	s.e.	Mean	s.e.	Mean	s.e.
**Place of living**								
*Days at home*	18.14	(0.03)	15.79	(0.03)	12.05	(0.03)	98.05	(0.17)
*Days in long-term institutions*	10.24	(0.03)	11.09	(0.03)	11.71	(0.03)	64.61	(0.19)
*Days in short-term institutions*	1.25	(0.01)	2.02	(0.01)	3.78	(0.02)	11.56	(0.06)
*Days in hospital*	1.19	(0.01)	2.43	(0.01)	4.29	(0.01)	12.70	(0.04)
**Healthcare use**								
Primary care								
*GP visits*	1.30	(0.00)	1.53	0.00	1.83	(0.01)	8.78	(0.02)
*Emergency primary healthcare visits*	0.49	(0.00)	0.56	0.00	0.56	(0.00)	3.16	(0.01)
Secondary care								
*Inpatient treatments*	0.25	(0.00)	0.40	0.00	0.85	(0.00)	2.42	(0.01)
*Outpatient treatments*	0.49	(0.00)	0.56	0.00	0.56	(0.00)	3.16	(0.01)
Home- and community-based care								
*Practical assistance hours*	2.95	(0.05)	2.96	0.05	2.85	(0.05)	17.63	(0.30)
*Nursing assistance hours*	8.83	(0.07)	9.56	0.07	10.24	(0.07)	55.84	(0.40)
**Healthcare costs (all ages)**								
*Primary*	117	(0)	186	(1)	451	(2)	1.174	(3)
*Secondary*	2.168	(75)	2.642	(14)	4.168	(23)	15.956	(51)
*Home- and community-based*	1.959	(4)	4.989	(12)	13.181	(36)	29.036	(71)
*Total*	4.244	(10)	7.816	(17)	17.801	(41)	46.166	(79)
**Healthcare costs (< 80 years)**								
*Primary*	136	(1)	216	(1)	513	(3)	1 354	(6)
*Secondary*	3 070	(20)	4 102	(28)	6 566	(47)	23 981	(103)
*Home- and community-based*	1 275	(7)	2 887	(20)	7 118	(56)	16 718	(112)
*Total*	4 482	(20)	7 204	(34)	14 198	(72)	42 053	(147)
**Healthcare costs (≥ 80 years)**								
*Primary*	104	(0)	164	(1)	407	(2)	1.047	(4)
*Secondary*	1.527	(8)	1.603	(11)	2.463	(18)	10.249	(40)
*Home- and community-based*	2.445	(5)	6.484	(15)	17.493	(44)	37.796	(83)
*Total*	4.075	(8)	8.251	(17)	20.363	(46)	49.091	(83)

#### Place of living

Based on data from NPR [22] and IPLOS [24], we estimated how many days individuals spent at home, in care institutions—including short-term care and long-term care institutions (i.e., nursing homes, sheltered housing, other round-the-clock care, and sheltered housing with 24-hour care)—and in hospitals during their last six months of life. The number of days at home was estimated by subtracting days in hospitals and in care institutions from 186 days, which corresponds to six months. We allowed days in hospitals and in care institutions to overlap, since patients who receive treatment in hospitals often keep their place in their long-term care institution.

### Statistical analysis

We used descriptive statistics to summarise the average healthcare use and costs during individuals’ last six months of life. We present both total healthcare use and costs by the following time periods: all six months before death (total), as well as 6 to 4 months, 3 to 2 months, and 1 month before death^1^. To enable comparison between time periods, we present healthcare use and costs as average resource use and costs per month for all time periods^2^. We present results for all decedents as well as stratified by cause of death. For all causes of death, we describe healthcare use and costs separately for those aged older than 80 years and for those younger than 80 years at the time of death. We provide supplementary materials with detailed cause-specific healthcare use and costs at all levels of formal care for the time periods 6 to 4 months, 3 to 2 months, and 1 month before death for all decedents (Supplementary Material 1), for those aged younger than 80 years (Supplementary Material 2 & 4) and for those aged 80 years or older (Supplementary Material 3 & 4). To estimate relevant healthcare use for other countries or contexts, our variables on resource use can be multiplied by country- or context-specific unit costs.

## Results

Between 2009 and 2013, a total of 207,299 individuals died in Norway, or approximately 41,000 individuals per year. The majority of those who died were older than 80 years at the time of death (Table 3). We list the categories of underlying cause of death in order of prevalence: Diseases of the circulatory system (31%), Cancer (26%), Diseases of the respiratory system (10%), Injuries (6%), Mental and behavioural diseases (5%), Diseases of the nervous system and sense organs (4%), Diseases of the digestive system (3%), Endocrine, nutritional, and metabolic diseases (2%), Communicable diseases (2%), and Other diseases (10%). Dementia was the most common underlying cause of death in both Mental and behavioural diseases (Unspecified dementia 77% + Vascular dementia 8%) and Diseases of the nervous system and sense organs (Alzheimer’s disease 45%) (Table 1). The most common causes of deaths in the other categories can be viewed in Table 1.

**Table 3:** Descriptive statistics of all decedents and by cause of death. Numbers given as number of individuals (n) and proportion of population (p)

		All decedents		Communicable (A00-B99)		Cancer (C00-97)		EMD^1^ (E00-99)		Mental (F00-99)		Nervous (G00-H95)		Circulatory (I00-99)		Respiratory (J00-99)		Digestive (K00-93)		Injury (V01-Y89)		Others (L-U)	
		*n* = 207 299		*n* = 4 845		*n* = 53 915		*n* = 5 147		*n* = 10 419		*n* = 8 394		*n* = 65 027		*n* = 20 395		*n* = 6 379		*n* = 12 729		*n* = 20 049	
**Age at death**																							
		n	p	n	p	n	p	n	p	n	p	n	p	n	p	n	p	n	p	n	p	n	p
< 50		10 694	(0,05)	135	(0,03)	2 363	(0,04)	285	(0,06)	216	(0,02)	459	(0,05)	1 109	(0,02)	201	(0,01)	194	(0,03)	4 009	(0,31)	1 723	(0,09)
50–59		11 721	(0,06)	177	(0,04)	4 935	(0,09)	262	(0,05)	328	(0,03)	430	(0,05)	2 120	(0,03)	513	(0,03)	493	(0,08)	1 437	(0,11)	1 026	(0,05)
60–64		10 829	(0,05)	134	(0,03)	5 099	(0,09)	207	(0,04)	217	(0,02)	391	(0,05)	2 185	(0,03)	694	(0,03)	402	(0,06)	610	(0,05)	890	(0,04)
65–69		14 279	(0,07)	227	(0,05)	6 648	(0,12)	321	(0,06)	269	(0,03)	521	(0,06)	3 149	(0,05)	1 195	(0,06)	428	(0,07)	495	(0,04)	1 026	(0,05)
70–74		15 995	(0,08)	253	(0,05)	6 623	(0,12)	372	(0,07)	367	(0,04)	702	(0,08)	3 972	(0,06)	1 658	(0,08)	474	(0,07)	456	(0,04)	1 118	(0,06)
75–79		22 634	(0,11)	524	(0,11)	7 683	(0,14)	537	(0,10)	723	(0,07)	1 103	(0,13)	6 550	(0,10)	2 512	(0,12)	688	(0,11)	690	(0,05)	1 624	(0,08)
80–84		34 345	(0,17)	878	(0,18)	8 886	(0,16)	816	(0,16)	1 621	(0,16)	1 580	(0,19)	11 415	(0,18)	4 035	(0,20)	1 032	(0,16)	1 188	(0,09)	2 894	(0,14)
85–89		42 374	(0,20)	1 262	(0,26)	7 241	(0,13)	1 119	(0,22)	2 855	(0,27)	1 789	(0,21)	16 339	(0,25)	4 618	(0,23)	1 364	(0,21)	1 786	(0,14)	4 001	(0,20)
> 90		44 428	(0,21)	1 255	(0,26)	4 437	(0,08)	1 228	(0,24)	3 823	(0,37)	1 419	(0,17)	18 188	(0,28)	4 969	(0,24)	1 304	(0,20)	2 058	(0,16)	5 747	(0,29)
**Sex (females)**		107 393	(0,52)	2 683	(0,55)	25 108	(0,47)	2 852	(0,55)	6 775	(0,65)	4 826	(0,57)	34 934	(0,54)	10 583	(0,52)	3 583	(0,56)	5 290	(0,42)	10 759	(0,54)

^*1*^ *EMD = Endocrine, nutritional and metabolic diseases*

### Healthcare use and costs

#### All decedents

For the 207,299 decedents, the average healthcare costs per individual in the last six months of life was €46,166. The majority of healthcare resources were used in home- and community-based care (63%), followed by secondary care (35%) and primary care (2%). As death approached, healthcare use increased across all levels of care. On average, individuals used €17,801 in the last month of life, compared to €7,816 per month in the 3 to 2 months before death and €4,244 per month in the 6 to 4 months before death (Table 2). During their last six months of life, individuals spent most days at home (52%) and in care institutions (41%), and the fewest days in hospital (7%) (Table 2). The number of days individuals spent at home per month decreased as death approached (-6 days) (Table 2); correspondingly, the average number of days individuals spent in care institutions (+ 4 days) and at the hospital (+ 3 days) increased in the same time (Table 2). 

On average, individuals received 2 inpatient and 3 outpatient treatments, visited their GP 9 times and had 3 emergency primary healthcare visits during their last six months of life (Table 2). They received 18 h of practical assistance and 56 h of nursing assistance during their last six months of life (Table 2). Similar to costs, healthcare use increased as death approached.

#### By cause of death

**Fig. 1:**
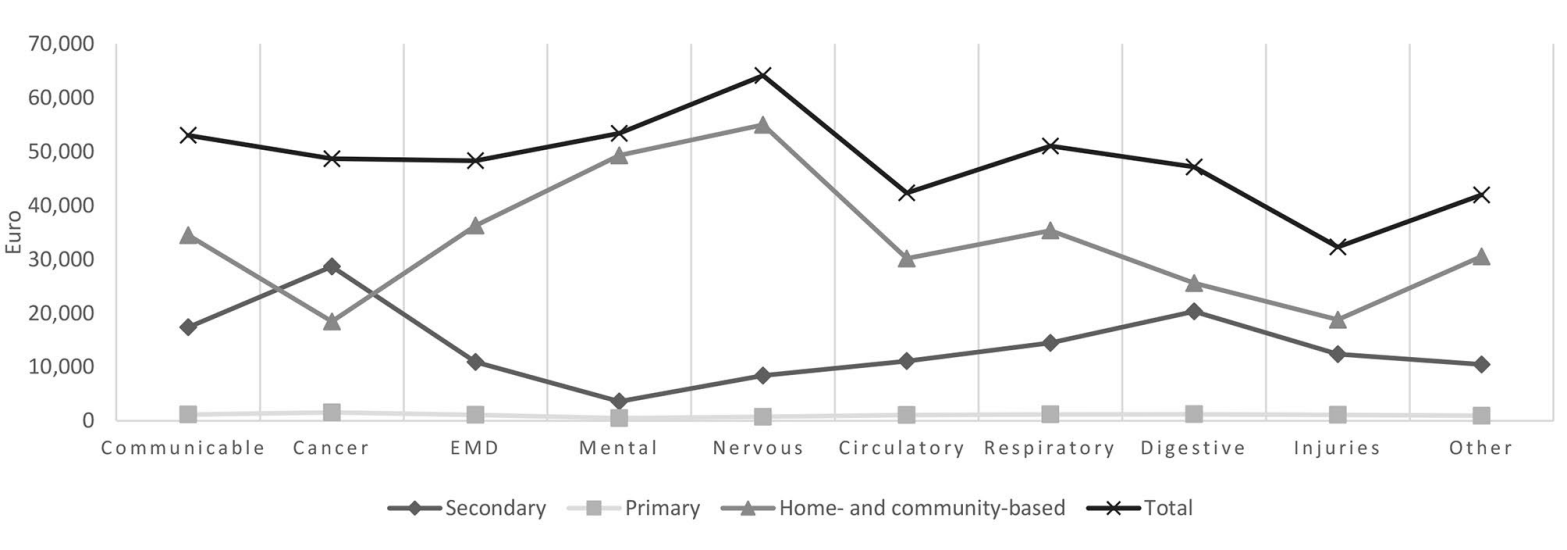
Total healthcare costs by level of care and cause of death

Average total healthcare costs in the last six months of life varied by cause of death, ranging from €32,276 (Injuries) to €64,123 (Diseases of the nervous system) (Fig. 1). Costs were lowest in primary care and highest in home- and community-based care for all causes of death except cancer, for which costs were highest in secondary care (Fig. 1). Individuals used different healthcare services depending on their cause of death. For example, individuals dying with endocrine/nutritional/metabolic diseases and individuals dying with cancer both used on average approximately €48,000 in the last six months of life; however, if total costs are decomposed by care level, it can be seen that cancer patients used more than twice as much in secondary care (€28,655) compared to individuals with endocrine/nutritional/metabolic diseases (€10,931), who in turn used twice as many resources in home- and community-based care (€36,262) compared to cancer patients (€18,454, Fig. 1 & Supplementary Material 1). Individuals dying with mental and nervous diseases, mostly dementia, received 86–92% of their care in the last six months of life outside secondary care, mostly in home- and community-based care. In contrast to individuals with dementia, individuals with digestive diseases or injuries used less resources in home- and community-based care, 38% and 58% respectively (Supplementary Material 1).

**Fig. 2:**
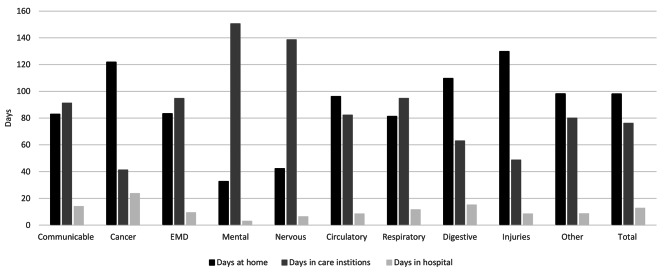
Place of living in the last six months of life by cause of death

Place of living differed by cause of death. While individuals dying with communicable diseases, circulatory diseases, digestive diseases, injuries, or other diseases spent most days at home, individuals dying with mental and nervous diseases spent most days in care institutions. The number of days in hospital in the last six months of life varied considerably, from 3 days in hospital for patients with dementia to 24 days in hospital for cancer patients (Fig. 2 & Supplementary Material 1). Individuals with communicable diseases, respiratory diseases, and digestive diseases spent 12 to 15 days in hospital, while individuals with endocrine/nutritional/metabolic diseases, nervous diseases, circulatory diseases, and injuries spent 6 to 9 days in the hospital in the last six months of life (Fig. 2 & Supplementary Material 1).

Individuals dying with nervous diseases, including Parkinson’s and Alzheimer’s disease, used more practical (72 h) and nursing (110 h) assistance than those dying from other causes of death (Supplementary Material 1). The amount of nursing assistance received by individuals with injuries was the lowest, at 15 h, while cancer patients received the least practical assistance, at 10 h (Supplementary Material 1). On average, individuals with cancer received the highest number of inpatient, outpatient treatments and GP consultations, while individuals with mental and nervous diseases had the fewest (Supplementary Material 1).

**Fig. 3:**
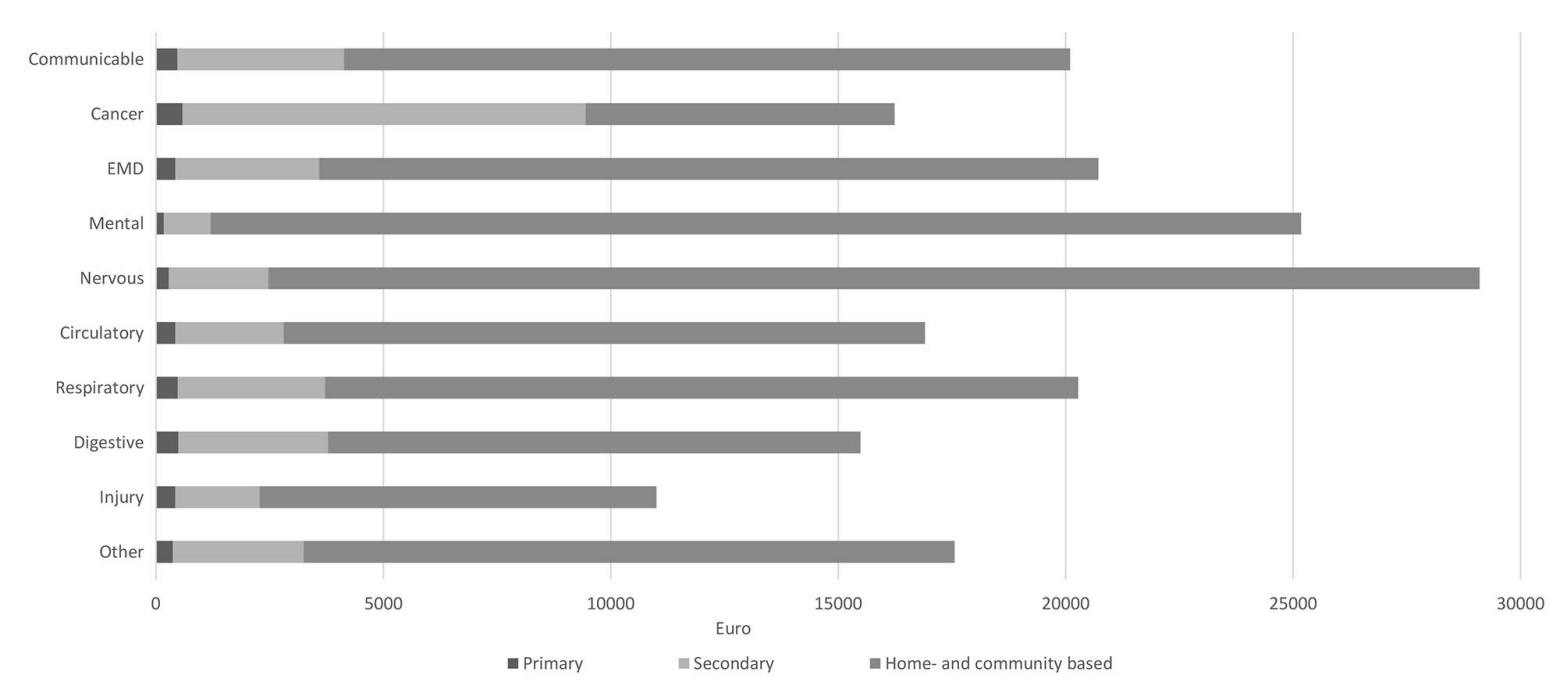
Healthcare costs in the last month of life by level of care and cause of death

Compared to the average cost in the last month of life (€17,800; Table 2), higher costs were observed for those dying with communicable, mental, nervous, endocrine/nutritional/metabolic, and respiratory diseases (Fig. 3, Supplementary Material 1). In the last month of life, dying with nervous diseases was associated with the highest average costs (€29,000), while the lowest costs were observed for those dying with injuries (€11,000) (Fig. 3, Supplementary Material 1). For individuals dying with all causes except cancer, home- and community-based care constituted approximately 80% of care in the last month of life. For individuals dying with mental and nervous diseases, 91–95% of care in the last month of life was provided through home- and community-based care (Fig. 3, Supplementary Material 1). For detailed estimates of healthcare use and costs for all levels of care, for all causes of death and for all age groups, we refer to our comprehensive Supplementary Materials.

#### By age

The total healthcare cost during the last six months of life for individuals who died before the age of 80 years was €42,053, with these costs distributed as follows: 40% in home- and community-based care, 57% in secondary care, and 3% in primary care (Table 2, Supplementary Material 2). For an individual who died at the age of 80 years or older, average total healthcare costs accumulated to €49,901, with 79% spent in home- and community-based care, 21% in secondary care, and 2% in primary care (Table 2, Supplementary Material 3). Home- and community-based care was the dominant form of care for those aged 80 years and older, regardless of the cause of death (Table 2, Supplementary Material 3 & 4). However, among those younger than 80 years, the level of care varied depending on the cause of death (Supplementary Material 2 & 4). For instance, for those aged  80 years or older, the proportion of overall expenses allocated to home- and community-based care ranged from 54% for individuals with cancer to 94% for individuals with mental and nervous diseases, mostly dementia (Supplementary Material 3). However, for those aged  younger than 80 years at time of death, this proportion ranged from 25% (cancer) to 83% (mental and nervous diseases) (Supplementary Material 2 & 4). We provide comparable data for all causes of death by age (Supplementary Material 2–3), including a figure comparing age groups (Supplementary Material 4).

## Discussion

Healthcare use and costs differed by level of care, cause of death, age at death, and time to death. For all individuals who died in Norway between 2009 and 2013, the average total cost was €46,000 in the last six months of life. For all decedents, the majority of healthcare resources in the last six months of life were used at the level of home- and community-based care (63%, Fig. 1; Table 2). Whether most care was utilised in home- and community-based or secondary care differed by cause of death and by age (Supplementary Material 1–4). Those who died aged 80 years or older used most home- and community-based care across all causes of death (Supplementary Material 3 & 4). For those who died being younger than 80 years, the predominance of home- and community-based care was only true for individuals dying with mental and nervous diseases (Supplementary Material 2 & 4).

For all decedents, across all age groups, resource use increased, the shorter the time to death (Table 2, Supplementary Material 1). On average, the last four weeks of life accounted for one third of all health care costs incurred in the last six months of life (Table 2). The costs associated with dying from injuries, circulatory diseases, and other diseases were lower than the average costs during the last six months of life, most likely due to sudden death (Supplementary Material 1). In contrast, individuals who died from mental and nervous diseases, communicable diseases, and respiratory diseases were more likely to have received care for a longer period of time before death, resulting in higher-than-average healthcare costs in the last months of life. Individuals dying with cancer, digestive diseases, and endocrine/nutritional/metabolic diseases had close to average costs during the last six months of life (Supplementary Material 1 & 4).

Our findings have important implications for decision-makers who are responsible for resource allocation in healthcare, as well as for healthcare planners who have to anticipate future healthcare needs. In the future, improved survival from some diseases will likely shift the causes of death of at the population level; for example, if improvements in cancer treatment prevent cancer-related deaths, more individuals will die from other diseases later in life rather than from cancer. Our analysis provides knowledge on resource use and costs associated with diseases beyond cancer which are common in older age, such as dementia. Dementia is currently the seventh-leading cause of death worldwide, and its prevalence is expected to double every 20 years [19, 32]. Dementia is estimated to be one of the costliest diseases globally [33].

Kinge and colleagues estimated that dementia was the disease with the highest health spending, at 10.2% of total national health spending in Norway already in 2019 [30]. Evidence which facilitates assessment of the cost-effectiveness of new dementia drugs and which helps in planning the expected need for relevant healthcare is urgently needed around the world. We found that individuals with dementia used an above-average amount of healthcare resources in the last six months of life and that approximately 90% of these resources were used in home-and community-based care. These findings are in line with a 2023 Norwegian population-based registry study, which revealed that 78% of healthcare expenses related to dementia were spent on nursing homes [30]. Similarly, a systematic review summarized that individuals with dementia used more resources for professional home care and for nursing facilitates compared to individuals suffering from other diseases [18]. This type of cause-specific evidence can help healthcare planners prepare for future demands.

The validity of a decision-analytic model depends on the validity of the data used to populate the model. In the absence of cause-specific estimates on resource use and costs, modellers habitually use proxy parameters, which are available in the existing literature, or generic unit costs. Our study indicates that using proxy data from other disease types can be problematic: if cancer patients’ resource use is utilised to model resource use for dementia patients, this will systematically bias results—particularly the share of resource use taken up by home- and community-based care (38% for cancer patients vs. 92% for dementia patients) (Supplementary Material 1-4). Modellers should always strive to provide a complete picture of relevant disease pathways and to include the real-world economic burden of care at all levels for the entire lifespan [34]. Currently, due to gaps in knowledge regarding healthcare usage and costs, this is not feasible for all patient groups. Our findings enable the use of cause-specific estimates instead of proxy parameters, which has the potential to enhance estimates of resource use, models, and thus decisions allocating healthcare resources in various settings.

Previous studies on resource use and costs in the last months of life have often focused selectively on single causes of death and specific care variables, mainly secondary care variables. Methodological differences in samples, time frames, and healthcare settings make it difficult to compare parameters across studies. It is not possible to explain the variance in healthcare use and costs between previous studies and our findings based on the descriptive analyses we performed; nevertheless, it is helpful to put our findings into context. In the following, we focus solely on dementia, as it would be overwhelming to discuss findings for all causes of death.

The PAID 3.0, a Dutch tool initially created to incorporate future disease costs in economic evaluations, offers annual healthcare costs from the Netherlands, stratified by ICD-10 codes, age, and time to death [35]. This data is based on Dutch cost-of-illness data published in 2017 [36]. In the last year of life, the total average healthcare cost for individuals with mental and behavioural diseases (F00–99) was estimated with PAID to be €57,018 [35]. When we adjust our total cost estimate for mental diseases from 2013 to 2017, the two estimates are very similar (PAID: €57,018 vs. €58,736). The same is true for secondary care costs for individuals with mental diseases (PAID: €11,192 vs. €12,025), while for home- and community-based care, the Dutch estimate is higher than our findings (PAID: €45,826 vs. €39,891). The PAID data is based on the entire last year of life, while our findings summarize costs for the last six months of life; however, since the majority of healthcare costs occur when death approaches, we consider the comparison with PAID data to be valuable, despite the different time frames.

In a recently-published systematic review, Sontheimer and colleagues examined the costs of dementia from the time of diagnosis until death across different studies [18]. They found significant variation in total cost estimates, ranging from €1385 per person for 104 dementia patients in Argentina [37] to €48,655 per person for 541 dementia patients in residential care in Australia [38]. This wide range emphasises the importance of studies (like ours) which estimate healthcare costs in a common methodological framework. The reviewed studies support our finding that individuals with dementia receive most care through home- and community-based care: Patients with dementia had significantly higher costs for nursing facilities and professional home care for than patients without dementia. Interestingly, the total costs for inpatient and outpatient treatments were similar for patients with and without dementia. This finding supports our conclusion that the additional burden associated with dementia, compared to other causes of death, arises from demand in home and community-based care. This highlights the importance of reflecting healthcare use and costs from home and community-based care in decision analytic models.

Our findings might raise the question of whether our grouping of causes of death was detailed enough. For data anonymity reasons, the grouping of decedents into these categories of cause of death was predefined by the registries before the data were delivered to the researchers. We are nevertheless confident with the present grouping, since the categories of cause of death in this analysis cover the major causes of deaths and provide a wider range of causes of death than commonly seen in previous studies. An earlier study estimated healthcare use and costs for individuals dying with different types of cancer and showed that the specific cancer was less influential than other factors, such as individuals’ age and access to informal care [12]; whether this is true for subgroups for other causes of death could not be assessed with our dataset and thus remains largely unknown.

### Generalisability

Some aspects regarding the generalisability of our findings must be discussed. First, our data come from 2009 to 2013; this time delay occurred because it took years to obtain access to comprehensive registry data. Since that period, several changes might have influenced individuals’ healthcare use in the last months of life. For instance, life-prolonging treatments might have increased survival, and patients who die today might differ from those who died in 2009–2013. Individuals dying today might be older, or they might die from different causes which can influence healthcare use. In addition, societal changes might have shifted individuals’ healthcare use. Importantly, Norway (along with other countries) is increasingly encouraging the shifting of treatment from secondary care to more local levels (i.e., the municipality); consequently, patients are meant to spend less time in hospitals, while stays in municipal care institutions are likely to increase. New analyses on updated data are needed in order to evaluate whether this has happened. To our knowledge, our estimates are currently the most comprehensive and updated with regard to resource use and costs for all decedents and for all causes of death.

Second, our findings can be generalised to settings which are similar to Norway, where healthcare is universally covered, out-of-pocket-payments are relatively low, and it is common to use formal care at the end of life. In healthcare settings with differences in incidence and severity of diseases, available healthcare resources, clinical practices, and relative price levels, our findings on healthcare use can still be informative [39]. To facilitate the adaptation of our results to other countries, we have reported our results for healthcare use and costs separately in the Supplementary Materials 1–4. This enables readers to multiply our estimates on healthcare use with any other country-specific unit costs.

Third, we are aware that informal caregivers carry a considerable burden when individuals approach the end of their lives [40, 41]. Cultural differences with regard to how much informal care families provide during this period will influence findings reporting the use of formal healthcare. In a study evaluating the number of individuals who died at home, Cohen and colleagues (2010) found that home death for persons dying with cancer varied from 12.8% in Norway to 22% in England, 23% in Wales, 28% in Belgium, 36% in Italy, and 45% in the Netherlands [42]. In 2022, 15% of all those who died from cancer in Norway died in private homes [21]. Place of death is likely connected to where individuals receive care; consequently, the amount of informal care and that of formal healthcare use might differ between these countries. In societies in which informal care is the dominant form of care in the last months of life, our findings can still be of interest, but they should be generalised with caution.

Finally, we consider it worth mentioning that it is challenging for physicians to identify the correct immediate cause of death. For this reason, we chose to use the underlying cause of death in our analysis. Still, using CDR as the source of cause of death has its limitations, primarily related to coding [43]: for example, there is a risk of different physicians coding multimorbid patients in different ways. We validated the underlying cause of death for all individuals with cancer by comparing the ICD-10 codes provided in CDR [21] with those in The Cancer Registry of Norway [44]. We found a reassuring overlap, which gives us confidence that CDR provided reliable information for all causes of death.

We report a comprehensive picture of the quantity of healthcare used during the last six months of life. At the same time, we acknowledge the relevance of assessing the quality of care. More research is needed to explore to what extent end-of-life care aligns with the preferences of patients and their next-of-kin. Unfortunately, our current dataset does not provide answers to these important questions, but we are optimistic that we can address them in future studies.

## Conclusion

Using comprehensive, population-based registry data, we described healthcare use and costs in the last six months of life by level of care, for all decedents and stratified by ten major ICD-10 categories summarising all causes of death. Our research shows that healthcare use and costs in the last six months of life differ depending on cause of death: The total amount of healthcare varies, as does the level of care at which most resources were utilised (primary, secondary, or home- and community-based care). These findings enable decision-makers to make more informed decisions about recource allocation and healthcare planners to better anticipate future healthcare needs.

### Electronic supplementary material

Below is the link to the electronic supplementary material.

Supplementary Material 1: All decedents by all causes fo death

Supplementary Material 2: Decedents younger than 80 years by all causes of death

Supplementary Material 3: Decedents older than 80 years by all causes of death

Supplementary Material 4: Comparing healthcare costs by age at death

## Data Availability

Legal restrictions apply to the availability of the data underpinning the findings of this study, which were used under license for the current study. The data is not available upon request from the authors, and it cannot be made available to referees, editors, or readers upon request.

## Footnotes

1 To preserve anonymity, we did not receive data for shorter time periods from the registries.

2 We have divided the numbers for the 3-month periods (e.g., 6-4 months before death) by 3 to obtain monthly estimates. For the entire analysis, we assumed no healthcare use for missing registrations.

## Abbreviations

CDR: The Norwegian Causes of Death Register
DRG: Diagnosis-related group
ICD: International Classification of Diseases
GP: General Practitioner
IPLOS: The Individual-based Statistics for Nursing and Care Services Register
KOSTRA: The Municipality-State-Reporting
KUHR: Norwegian Control and Payment of Health Reimbursements Database
NPR: The Norwegian Patient Register
WHO: World Health Organisation
