# Supplementary material for: Healthcare use and costs in the last six months of life by level of care and cause of death
Source: BMC Health Serv Res. 2024 May 30;24:688. doi: 10.1186/s12913-024-10877-5 (PMC11140868; doi:10.1186/s12913-024-10877-5)
Supplement: Supplementary file 4 — Supplementary Material 4: Comparing healthcare costs by age at death [file 12913_2024_10877_MOESM4_ESM.docx]

*Appendix 4: Comparative figures on healthcare costs by age*

*Appendix 4a: Healthcare costs for those younger than 80 years by level of care and cause of death*


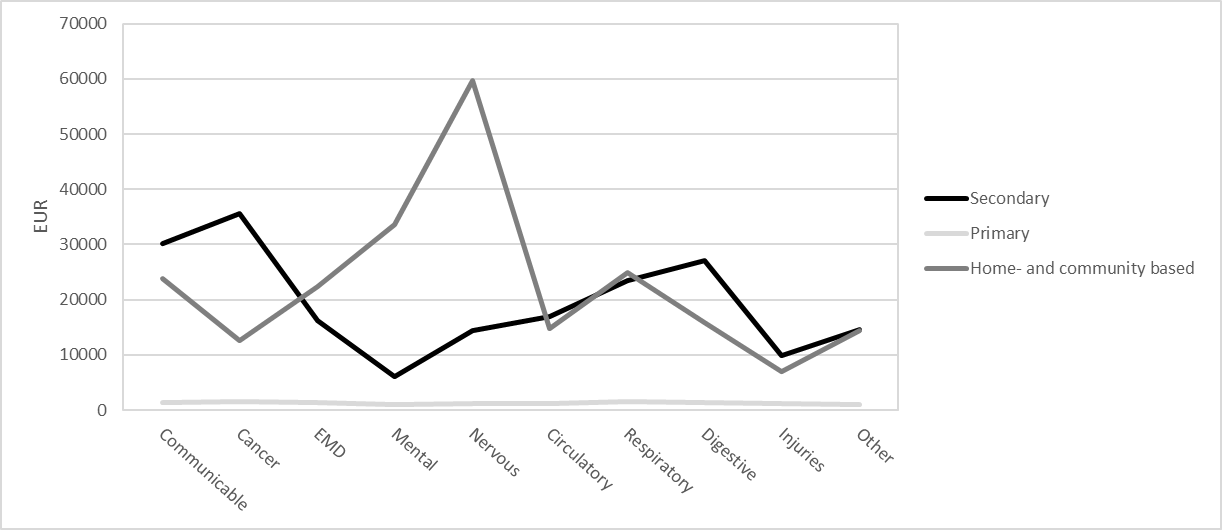


*Appendix 4b: Healthcare costs for those 80 years or older by level of care and cause of death*
